# Supplementary material for: Genome-Wide Association Mapping of Prostrate/Erect Growth Habit in Winter Durum Wheat
Source: Int J Mol Sci. 2020 Jan 8;21(2):394. doi: 10.3390/ijms21020394 (PMC7014441; doi:10.3390/ijms21020394)

Supplemental Figure 1. Population structure of the whole collection using 30,611 DArT-seq markers.

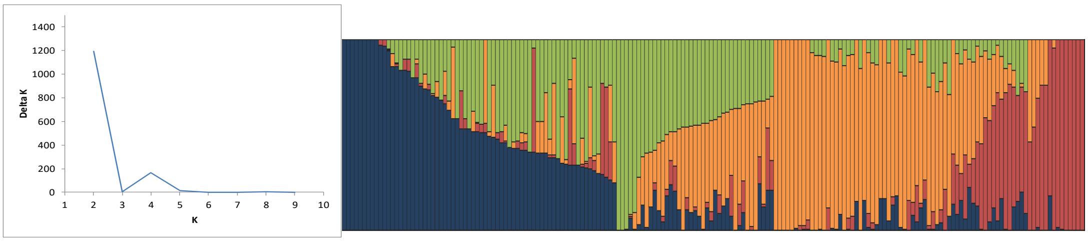

Supplemental Figure 2. DAPC results: (A) Number of clusters (4) at the lower BIC value; (B) Individuals in red attributed to each of the four groups; (C) Number of components to be retained for this analysis (3).

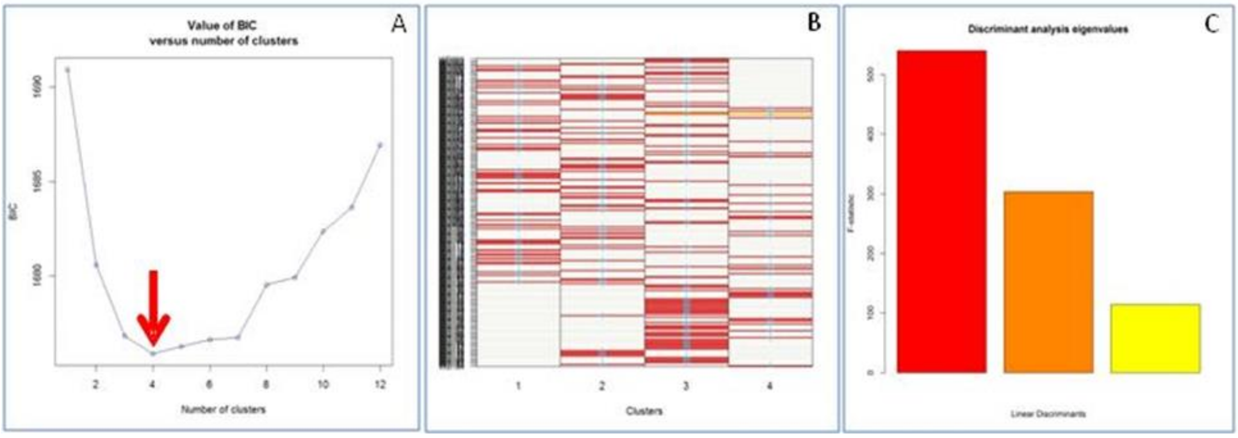

Supplement: Supplementary file 1 [file ijms-21-00394-s001.zip › ijms-674432-SI.pdf]
